# Trust is in the eye of the beholder: How perceptions of local diversity and segregation shape social cohesion

**DOI:** 10.3389/fpsyg.2022.1036646

**Published:** 2023-01-16

**Authors:** Jasper Van Assche, Sofia Ardaya Velarde, Alain Van Hiel, Arne Roets

**Affiliations:** ^1^Department of Developmental, Personality and Social Psychology, Ghent University, Ghent, Belgium; ^2^Center for Social and Cultural Psychology (CESCUP), Université Libre de Bruxelles, Brussels, Belgium

**Keywords:** ethnic-cultural diversity, local segregation, perceptions, trust, social cohesion

## Abstract

A more nuanced understanding of the complex relationship between ethnic diversity and social cohesion is needed. Ever since Robert Putnam (2007) has put forward the highly contested constrict claim holding that diversity is related to less trust and more social withdrawing, hundreds of follow-up studies across the globe have been conducted. In the present contribution, we investigated the association between diversity and “hunkering down” in the Netherlands, hereby taking into account the role of segregation. Indeed, Uslaner (2012) pointed to local segregation as the true motor of the so-called diversity effects on intergroup relations in general, and trust in others in particular. We did not only investigate objective indicators of diversity and segregation, but also added an “eye of the beholder” perspective by probing into the subjective perceptions of these variables. Specifically, in a stratified community sample of 680 Dutch ethnic-cultural majority members (52% male, mean age 51), we assessed the additive and interactive effects of four variables (objective diversity, perceived diversity, objective segregation, and perceived segregation) at the municipal level in the prediction of three outcomes (generalized trust, ingroup trust, and outgroup trust). The results revealed three interesting patterns. First, neither of the objective indicators of diversity and segregation, nor their interaction effect significantly predicted any type of trust. Second, higher perceptions of diversity and higher perceptions of segregation were negatively associated with outgroup trust (but not with generalized and ingroup trust). Third, and most importantly, there was a significant interaction effect between perceived diversity and perceived segregation, indicating that simultaneous perceptions of high levels of diversity and high levels of segregation were related to the lowest levels of trust in other ethnic-cultural groups. These findings shed a more nuanced light on the diversity debate, showing that perceptions of segregation shape diversity effects. In sum, the present study shows that perceived rather than objective indicators of diversity and segregation matter, and that both diversity and segregation should be taken into account when it comes to social cohesion in general, and trust in particular.

## Introduction

According to Robert D. Putnam’s constrict claim (2007), ethnic diversity makes people “hunker down - that is, to pull in like a turtle” (p. 149). Using data from the 2000 Social Capital Community Benchmark Survey (SCCBS), with over 29,000 respondents in 41 United States communities, he put forward that higher diversity would be associated with less trust; in general, in ethnic-cultural outgroups, and even in one’s own ingroup (Putnam, 2007). Social cohesion (i.e., the extent of connectedness and solidarity among groups in society) and social capital (i.e., the networks of relationships among people who live and work in a particular area, enabling that local environment to function effectively) thus seem to be at stake in diverse environments. Such a dramatic conclusion can have massive implications for policy makers aiming at improving harmony in their local neighborhoods and cities (see Kearns, 2003). It is therefore not surprising that Putnam, a lifelong protagonist of the importance of social capital, was invited to the White House by then-president Obama to better understand “community in America” and to discuss how the presence of multiple ethnic and cultural groups can divide but also unite (White House Archives, 2013).

### A jumble of post-Putnam results

In the academic world alike, this controversial “hunkering down” hypothesis struck like a bomb and motivated many scholars to test and evaluate Putnam’s results (Hewstone, 2015). The results were mixed. Several studies looked at general trust. Early on, Leigh (2006) found a small, negative relationship between city-level diversity and general trust in Australia, as did Gundelach and Freitag (2014) later in Germany. However, Sturgis and Smith (2010) and Sturgis et al. (2011) failed to find such a relationship in the United kingdom, Ivarsflaten and Strømsnes (2013) found no such association in Norway, neither did Wallman Lundåsen and Wollebæk (2013) in Sweden, nor Morales and Echazarra (2013) in Spain. Tolsma et al. (2009) found no diversity-general trust link in the Netherlands, and Kazemipur (2006) even found a positive link between diversity and general trust in Canadian cities. Furthermore, a number of authors investigated outgroup trust in addition to general trust. For example, Robinson (2020) reported a positive association between diversity and outgroup trust in Malawi. Lancee and Dronkers (2008) collected a large Dutch sample and reported negative effects of diversity on general trust, but not on outgroup trust (later finding no diversity-outgroup trust relationship in another Dutch sample, see Lancee and Dronkers, 2011).

Indeed, recent narrative and meta-analytic reviews show that follow-up studies of Putnam (2007) revealed inconsistent results for the constrict claim. As Van Assche et al. (2019a) stated in their post-Putnam literature review, empirical findings on the correlates of diversity seem highly diverse themselves. This was echoed by no less than three meta-analytic accounts of this topic – which again points to its reputation and importance. Schaeffer (2014) reported substantial variation in the relationship between ethnic diversity and trust, with 48% of the reviewed studies finding a significant negative relationship, and 52% failing to do so. van der Meer and Tolsma (2014) even found that diversity (measured locally within countries) was significantly related to lower outgroup trust in only 9% of the studies, and it was related to lower ingroup trust in 20% of the reviewed cases. In an attempt to quantify this mixed evidence into meta estimates, Dinesen et al. (2020) projected partial correlations of diversity with general, ingroup, and outgroup trust of *r* = −0.02, *r* = −0.02, and *r* = −0.01, respectively.

In light of these mixed findings on the effects of actual diversity, several authors demonstrated the importance of distinguishing between actual and perceived diversity. Abascal and Baldassarri (2015) found no effects of actual diversity on general, ingroup, and outgroup trust in the U.S, and, in the same vein, Schmid et al. (2014) did not find effects of actual diversity on ingroup and outgroup trust in British neighborhoods. Importantly however, these authors revealed small negative associations of *perceived* diversity with both outcomes. Van Assche et al. (2016) similarly found that actual diversity across Dutch neighborhoods was not negatively related to outgroup trust, but perceived diversity was. An important takeaways that can be deduced so far is that perceptions seem to play a larger role than the actual proportion of other groups in the local area. The first aim of the current contribution is thus to examine the association of actual and perceived diversity with all three types of trust (general, ingroup, and outgroup trust), expecting no significant effects of actual diversity, and small, negative effects of perceived diversity, in particular on outgroup trust (as this trust aspect is targeting the group that contributes to the ethnic-cultural local composition, see Van Assche et al., 2016).

### A search for suitable moderators

The divergences in previous studies have led scholars within social-psychological and political sciences to introduce other relevant influences. Indeed, both individual experiences and community characteristics might influence how much people trust each other (Alesina and La Ferrara, 2002). At the individual level, several studies revealed that diversity might have a very differential impact depending on personal dispositions. Stolle et al. (2008), for instance, showed with Canadian and United States data that not everybody in ethnically diverse neighborhoods is equally sensitive to their environment. Resonating this finding, other work consistently revealed that the negative effects of actual as well as perceived diversity were primarily (and sometimes even exclusively) found among a people with right-wing ideological attitudes, such as high levels of conformity values (Fasel et al., 2013), dangerous-worldview beliefs (Sibley et al., 2013), authoritarianism (Velez and Lavine, 2017; Van Assche et al., 2018a) social dominance orientation (Van Assche et al., 2018b), and a general right-wing political self-placement (Karreth et al., 2015; Russo et al., 2019).

At the contextual level, policies and segregation moderated diversity effects in several studies. Multicultural and integration policies have been introduced as a moderator of diversity effects, where diversity did not lead to negative outcomes when local policies were inclusive (Kesler and Bloemraad, 2010). Potentially, one reason why these policies moderate diversity effects is that they reduce segregation. In line with this, Uslaner (2012a) proposed contextual segregation as the key factor accounting for negative diversity effects, maintaining that segregation drives down trust more than diversity does. His rationale is that residential segregation isolates people from those who may be of a different background. This isolation, in combination with relatively high proportions of outgroup members, would be the main reason for lowered trust in general, in outgroups, but also in one’s ingroup (Uslaner, 2006).

Attesting to the importance of segregation, Sturgis et al. (2014), having conducted their study in London, United Kingdom, and Rothwell (2012), focusing on United States cities, found that diversity was not related to general trust, whereas segregation was negatively associated with it. In contrast with these results, Musterd (2003) found no negative effects of segregation in the Netherlands, and Douds and Wu (2018) even reported a positive link between segregation and general trust in Texas. Notably, none of these scholars tested the diversity × segregation interaction. Laurence (2017) did, and discovered that diversity only negatively impacted general trust in more segregated cities. Individuals living in diverse but integrated communities did not experience a “trust-penalty.” Mirroring this finding, Robinson (2020) found that diversity was only detrimental to outgroup trust when ethnic-cultural groups were living spatially segregated.

As such, a second aim of the present study is to examine the role of segregation in the diversity-trust association, hereby including objective as well as perceived indicators of segregation. Research in the domain of inequality already showed that the perceived extent of inequality better predicts intergroup outcomes as opposed to objective inequality, as not everyone attends to environmental cues (e.g., local neighborhoods, social networks, etc.) to the same degree (Phillips et al., 2020). Echoing this reasoning, and similarly to how perceived diversity relates more closely to trust than actual diversity, we propose that perceived segregation could also matter more for trust than actual segregation. Following our first research aim, we again expect particularly strong effects on outgroup trust as opposed to general and ingroup trust. In other words, we hypothesize (a) that general and ingroup trust will not be impacted by diversity and segregation, (b) that perceptions of diversity and perceptions of segregation will be related to lower outgroup trust, and (c) that the lowest levels of outgroup trust will be found among those individuals that concurrently perceive higher local diversity and local segregation.

## Materials and methods

### Participants

Data collection took place in the Netherlands, a West-European country that has experienced a sharp increase in ethnic-cultural diversity over the past couple of years (Onraet et al., 2021), which in turn resulted in a tense political debates (Van Assche et al., 2019b). Moreover, it is a context where Putnam’s constrict theory has been frequently put to the test. Indeed, it is the most popular context of examination after the United States and the United States (van der Meer and Tolsma, 2014). For the purposes of this study, we considered Dutch ethnic-cultural majority members to be the ingroup, and non-Western ethnic-cultural minority members as the outgroup. This definition of both groups was provided as an instruction before the start of the survey, it closely matches the “common” representation of immigrant outgroups in the Netherlands (Mügge and Haar, 2016), and it covers the four most numerous groups (i.e., people with a Turkish, Moroccan, Surinamese, and Moluccan background; Centraal Bureau voor de Statistiek, 2015).

Data were collected *via* an online questionnaire that was distributed to Dutch national citizens by an independent ISO 26362-certified survey company. The final dataset comprised a nationally stratified sample (by age, gender, and education level) of 680^1^ respondents without migration background from the 50 largest cities in the Netherlands^2^ (*M*_age_ = 50.72, *SD*_age_ = 16.70; 49% women). Thirty-four percent of the respondents had completed only primary school, 40% had completed only high school, and 27% had a college or university degree. Annual gross household income showed a fairly normal distribution, with 9% earning less than €12,500, 13% between €12,500 and €26,000, 25% between €26,000 and €39,000, 22% between €39,000 and €65,000, and 9% earned more than €56,000. Twenty-two percent of the respondents chose the option “I do not want to disclose this information.” Fifty-six percent of the sample owned their house, with 20% living alone, 40% living with their partner without children, 27% with partner and children, 4% without partner with children, 6% living with their parents, and 3% living with others (e.g., a roommate or a sibling).

### Measures

#### Actual diversity

We assessed the percentage of non-Western ethnic-cultural minority members within a specific city as an objective indicator of diversity. We used the available data from the Dutch Central Bureau of Statistics (Centraal Bureau voor de Statistiek, 2015), indicating the number of individuals of non-Western origin per city, and we calculated the percentage as a function of the total number of registered inhabitants to get a measure of relative actual diversity (*M* = 16.76%, *SD* = 9.15%, MIN = 4.11% in Emmen, MAX = 37.34% in Rotterdam).

#### Perceived diversity

We used two items to assess subjectively perceived diversity in one’s city (see also Van Assche et al., 2014): “How many people of non-Western immigrant origin live in your city?” and “There is a high chance of meeting people of non-Western immigrant origin in my city.” Respondents answered using 7-point rating scales ranging from 1 (*none/ totally disagree*) to 7 (*a lot/totally agree*). Both items formed a reliable scale (*α* = 0.78), with *M* = 4.97 (*SD* = 1.42).

#### Actual segregation

We assessed the dissimilarity index based on the available minority proportion data at the neighborhood and city level (Centraal Bureau voor de Statistiek, 2015). The dissimilarity index is a measure of segregation that indicates the evenness with which ethnic-cultural majority and minority members are distributed across neighborhoods that make up a city (James and Taeuber, 1985). The index ranges from 0 to 100, where a score of 0 indicates minimal segregation (and maximal evenness, i.e., when all neighborhoods have the same relative number of minority and majority members as the city as a whole). In contrast, a score of 100 points to maximal segregation (and minimal evenness, i.e., when no majority and minority members share a common area of residence within the city; Massey and Denton, 1988). The segregation scores across the cities varied from fairly low (Amstelveen: 8.42) to fairly high (Rotterdam: 46.54), with *M* = 27.54, *SD* = 11.28.

#### Perceived segregation

The two items to measure subjectively perceived segregation in one’s city were “In your city, to what extent do native Dutch people and people of non-Western origin live together, or isolated in separate neighborhoods?” and “My city is structured in clusters, with some neighborhoods almost exclusively populated by people of the same ethnic background.” Respondents answered using 7-point rating scales ranging from 1 (*completely intermixed/totally disagree*) to 7 (*completely separated/totally agree*). Both items formed a reliable scale (*α* = 0.77, *M* = 3.40, *SD* = 1.43).

#### General trust

General trust was measured by three items from the European Social Survey (see European Social Survey Round 10 Data, 2020). The items read: “Overall, do you think you should be careful when dealing with people, or can you trust most people?,” “Do you think most people want to take advantage of you if they have the chance, or do most people try to be honest?,” and “Do you think most people only consider themselves, or try to help other people?” Respondents answered using 7-point scales ranging from 1 (*You cannot be too careful/ Most people take advantage/ Most people only consider themselves*) to 7 (*Most people can be trusted/ Most people are honest/Most people help other people*), yielding a reliable scale with *α* = 0.84; *M* = 4.19, *SD* = 1.15.

#### Ingroup trust

For ingroup trust, respondents answered to one item (“When you specifically think of people of Dutch origin, do you think most of them are to be trusted, or not to be trusted?),” anchored by 1 (*Most people cannot be trusted*) and 7 (*Most people can be trusted*; *M* = 4.52, *SD* = 1.07).

#### Outgroup trust

Finally, we assessed outgroup trust with one item: “When you specifically think of people of non-Western immigrant origin, do you think most of them are to be trusted, or not to be trusted?,” using the same anchors (*M* = 4.02, *SD* = 1.23).

## Results

### Preliminary analyses

Because of the nested structure of the data (i.e., respondents living within cities), we first investigated whether multilevel analyses were warranted for our variables, following the two-step procedure suggested by Gelman and Hill (2007). In a first step, we estimated empty (intercept-only) models, which provide insight into the variances in our outcomes at the individual and contextual levels. Taking into account the higher-level structure for general, ingroup, and outgroup trust did not significantly improve the goodness-of-fit statistics of each model (i.e., all changes in −2 * log-likelihood were *χ*^2^s(1) < 0.02, all *p*s > 0.89). In a second step, we calculated the intraclass correlations to explore if there was substantial between-level variance in the scores of our outcome variables. All intraclass correlations were extremely small (<0.002), which renders the use of multilevel modeling unnecessary.

Correlations between all variables of interest are provided in Table 1. First, it can be noticed that all measures of diversity and segregation are moderately positively associated with one another. Second, the relationships of diversity and segregation indices with the trust measures are weak and often not statistically significant, with three noticeable exceptions, namely the negative associations between (a) perceived segregation and general trust, (b) perceived diversity and outgroup trust, and (c) perceived segregation and outgroup trust. Finally, all trust facets are strongly positively related to one another.

**Table 1:** Correlations between all study variables.

Measure	1	2	3	4	5	6
1. Actual diversity						
2. Perceived diversity	0.39^***^					
3. Actual segregation	0.34^***^	0.14^***^				
4. Perceived segregation	0.22^***^	0.22^***^	0.15^***^			
5. General trust	−0.01	−0.06	0.02	−0.10^*^		
6. Ingroup trust	0.01	0.00	0.02	−0.01	0.78^***^	
7. Outgroup trust	0.01	−0.11^**^	0.04	−0.13^***^	0.72^***^	0.66^***^

*p < 0.05; **p < 0.01; ***p < 0.001.

### Main analyses

We conducted six stepwise regression analyses examining the role of diversity and segregation in various facets of trust. In a first set of analyses, we exclusively focused on actual diversity as a predictor. Models 1, 2, and 3 investigated the effects of actual diversity, actual segregation, perceived segregation, their two-way interactions, and the three-way interaction on general, ingroup, and outgroup trust, respectively. In a second set of analyses, we focused on perceived diversity as a predictor. Models 4, 5, and 6 investigated the effects of perceived diversity, actual segregation, perceived segregation, their two-way interactions, and the three-way interaction on general, ingroup, and outgroup trust, respectively^3^. In each analysis, the main effects of all predictors were examined in the first step, all interaction terms were added in the second step (Table 2).

**Table 2:** Standardized estimates of the models testing the additive and interactive effects of actual (Models 1–3) or perceived diversity (Models 4–6) and actual and perceived segregation in predicting general, ingroup, and outgroup trust.

Measure	Model 1: General trust		Model 2: Ingroup trust		Model 3: Outgroup trust	
	Step 1	Step 2	Step 1	Step 2	Step 1	Step 2
Actual diversity (AD)	0.00	0.01	0.01	0.01	0.02	−0.03
Actual segregation (AS)	0.03	0.02	0.02	0.00	0.05	0.05
Perceived segregation (PS)	−0.10^*^	−0.10^*^	−0.01	−0.01	−0.15^***^	−0.15^***^
AD × AS		−0.01		0.01		0.09
AD × PS		0.06		0.12^*^		0.03
AS × PS		−0.04		−0.06		−0.05
AD × AS × PS		0.01		−0.01		−0.01
	**Model 4: General trust**		**Model 5: Ingroup trust**		**Model 6: Outgroup trust**	
	**Step 1**	**Step 2**	**Step 1**	**Step 2**	**Step 1**	**Step 2**
Perceived diversity (PD)	−0.05	−0.05	0.00	−0.01	−0.09^*^	−0.11^**^
Actual segregation (AS)	0.04	0.04	0.02	0.00	0.07	0.07
Perceived segregation (PS)	−0.09^*^	−0.09^*^	−0.01	−0.03	−0.13^**^	−0.14^***^
PD × AS		0.03		0.07		0.02
PD × PS		−0.03		0.01		−0.08^*^
AS × PS		−0.02		−0.05		−0.04
PD × AS × PS		0.01		0.06		0.05

*p < 0.05; **p < 0.01; ***p < 0.001.

**Figure 1:**
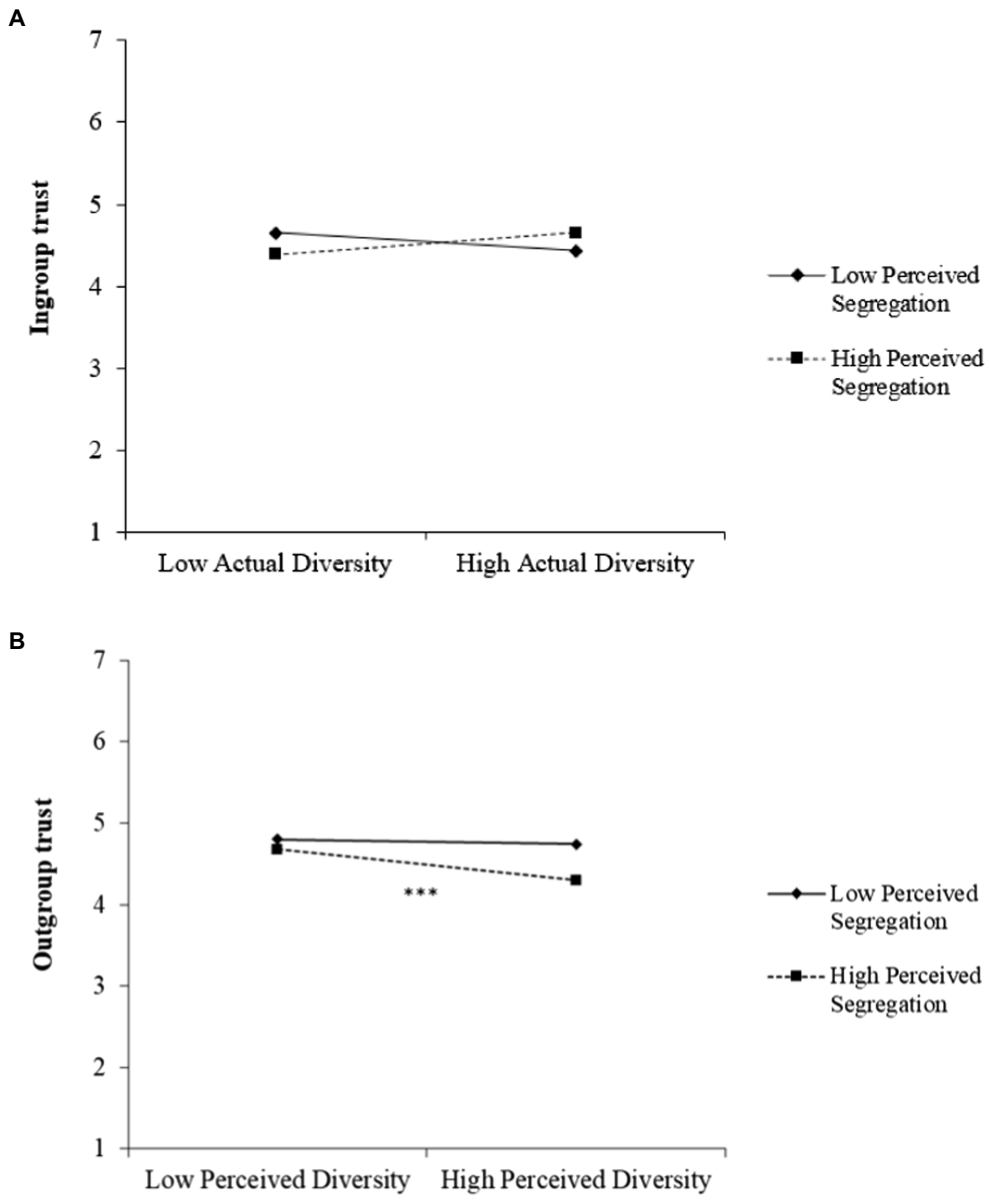
Interaction between actual diversity and perceived segregation on ingroup trust **(A)**, and between perceived diversity and perceived segregation on outgroup trust **(B)**.

Three interesting results emerged. First, as can be seen in Models 1 and 4, higher levels of perceived segregation are related to lower levels of *general* trust. Second, when it comes to *ingroup* trust (Models 2 and 5), no main effects of diversity and segregation are found, but there is a significant cross-over interaction effect between actual diversity and perceived segregation (see Figure 1A). Simple slope analyses show that actual diversity has a *negative*, yet not significant, effect on ingroup trust for those perceiving low levels of local segregation (*β* = −0.11, *p* = 0.11), whereas it has a *positive*, yet not significant, effect on ingroup trust for those perceiving high segregation (*β* = 0.12, *p* = 0.11). The highest levels of *ingroup* trust can thus be found in low-diverse cities among people perceiving low segregation, and in high-diverse cities among people perceiving a lot of segregation – although these differences are small and not statistically significant. Third, and most importantly, when it comes to *outgroup* trust (Models 3 and 6), negative main effects of perceived diversity and perceived segregation are found. Furthermore, there is a significant interaction effect between these two variables (see Figure 1B). In particular, simple slope analyses reveal that perceived diversity is not related to outgroup trust for those perceiving low levels of segregation (*β* = −0.05, *p* = 0.36), while it is clearly negatively related to outgroup trust among those high in perceived segregation (*β* = −0.20, *p* = 0.001). As a result, the lowest levels of *outgroup* trust can be found among those simultaneously perceiving high levels of diversity and segregation in their city.

## Discussion

The question of whether ethnic diversity affects communal social cohesion has become an increasingly prominent and contested topic of both political and academic debate (Sturgis et al., 2014). The work of Robert Putnam has been heavily popularized, leading to gloomy and even apocalyptic claims (Hallberg and Lund, 2005). Putnam himself (2007) used the colorful metaphor “diversity brings out the turtle in all of us” (p. 151). But do we all display lower trust when faced with a diverse environment? Recent research and reviews have called into question whether ethnic diversity *per se* has detrimental effects (Gereke et al., 2018), and also the empirical evidence from a variety of within- and cross-country studies lends only very qualified support to Putnam’s “hunkering down” hypothesis (Schaeffer, 2014; van der Meer and Tolsma, 2014). Calls for a critical research agenda were made (e.g., Hewstone, 2015; Dinesen and Sønderskov, 2018; Dinesen et al., 2020) to further this heated and unresolved debate and dismantle this “gordian knot” (Van Assche, 2019).

The present study adds nuance to the state-of-the-art by looking at the moderating influence of (actual and perceived) segregation in the relationship of (actual and perceived) diversity with general, ingroup and outgroup trust. First and foremost, we found no evidence to support Putnam (2007) pessimistic claim that diversity necessarily poses a challenge to social cohesion. Indeed, actual diversity was not related to any indicator of trust. Perceived diversity was also not related to general and ingroup trust. Yet, higher levels of perceived diversity were associated with lower levels of outgroup trust, but especially when paired with concurrent high levels of perceived segregation. Hence, living in a diverse area does not mean that one will display lower trust across the board. Even perceiving a great deal of city-level diversity does not automatically evoke lower trust in outgroup members, but people who perceive the distribution of minorities in their diverse city to be “uneven” will likely show lower outgroup trust.

### Perceived segregation as missing ingredient

One reason for this negative segregation effect can be found in the seminal work of Uslaner (2006, 2010, 2012a, 2012b, 2018). Uslaner (2006) posited that “it is not diversity that matters, it is how populations are distributed” (p. 3). Our findings shed an even more nuanced light on this argument. In particular, we found that it is not the actual distribution of a city that matters, it is how one *perceives* the proportion of outgroup members and the perceived evenness with which they are located in the city. As such, another quote of Uslaner (2012a), stating that “segregation, rather than diversity, lies at the root of low trust” (p. 244) deserves qualification. Indeed, we did not find that trust is lower in objectively diverse areas with large minority groups that are segregated from the majority groups. It is only outgroup trust that is affected, and only among individuals *perceiving* that ethnic-cultural minority members are numerous and living in separate neighborhoods than (ingroup) ethnic-cultural majority members. Hence, trust is in the eye of the beholder, and how we perceive the immediate world around us will likely determine whether we act like a turtle or not. Put differently, perceived segregation might well be the secret ingredient that was missing when delineating the effects of diversity on social cohesion.

The other significant interaction effect, between actual diversity and perceived segregation on ingroup trust, also deserves some attention. In diverse cities, ingroup trust seems to be highest among those perceiving a lot of local segregation. This is an interesting, yet unanticipated, result. We argue that perceptions of segregation might be used as some sort of ingroup-protective buffer by some majority members, where their neighborhood might be perceived as safer and less threatening (economically and culturally) when they have the idea that this neighborhood is mainly inhabited by fellow ingroup members, and outgroup members are mostly living in other areas of the city. Such a (thought) composition does not need to be in line with reality, but it could lead to ingroup members being trusted more. Of course, we should not overinterpret this finding, as the slope analyses showed that even for those perceiving a lot of segregation, the positive effect of actual diversity on ingroup trust is small and not statistically significant. Similarly, for those perceiving little segregation, the negative effect of actual diversity on ingroup trust is also small and not statistically significant. Nevertheless, future studies could specifically focus on this interaction effect to investigate if it might be qualified by other individual difference variables such as right-wing ideological attitudes (e.g., with the buffering mechanism of perceived segregation being more pronounced among those with more traditional political stances).

### Limitations and future research directions

Future studies could focus on other dimensions of social capital to further unravel the complex dynamics of diversity effects. In our research, we followed Putnam in his “mean and lean” definition of social capital, particularly tapping into trust facets, but we acknowledge that other dimensions (e.g., participation in local activities, formal volunteering, or informal help; see Gijsberts et al., 2012) can also be affected by the combination of diversity and segregation (and the perceptions thereof). Tapping into behavioral outcomes, using the so-called “lost wallet” vignette, is also an option that can serve as alternative means to measure social capital. Using the “lost wallet paradigm” in a large Dutch sample, Tolsma and van der Meer (2017) found that in more diverse municipalities, people were not less likely to believe that someone outside their neighborhood they did not know would return their wallet or purse with valuable items in case it got lost. It could be interesting to simultaneously examine the effects of actual municipal segregation and of perceptions of the diversity and segregation in respondents’ local environment on this behavioral intention. Finally, social cohesion in local areas can further impact intergroup relations. Indeed, in a multi-country Europe-wide study, Pellegrini et al. (2021) showed that lower trust significantly relates to higher anti-immigrant attitudes. As such, the “poisonous cocktail” of perceiving high levels of diversity and segregation in one’s city could not only be linked with lower trust in outgroups, but such lowered trust might further relate to more prejudice. We were able to tentatively test this moderated mediation model using Hayes’ Process macro (Model 7; Hayes, 2013) and found initial evidence for this pattern of results (see Appendix A for more detailed information). Future studies could elaborate on this empirical model by including other intergroup-related attitudes.

A second pathway for future studies is to replicate our results at different levels of analysis. Here, we focused on city-level diversity and separation of certain groups across neighborhoods in this city. Future studies could focus on more fine-grained (e.g., segregation across streets in a neighborhood) or broader levels of analyses (e.g., provinces within countries), each time taking into account how residents perceive the diversity and segregation in those areas. Effects at a larger scale level are not necessarily the same as effects at the lower level (Murie and Musterd, 2004). Studies at a very low (e.g., street) level are generally lacking (but see Dinesen and Sønderskov, 2015, for a notable exception). At a higher level, looking at countries, evidence for diversity effects again tends to be rather mixed, as was also revealed in the review of Dinesen et al. (2020). Kokkonen et al. (2014), for example, found negative effects of diversity, Hooghe et al. (2009) found no effects on general trust, while Gerritsen and Lubbers (2010) and Gundelach (2014) found positive effects of diversity on different facets of trust. Hence, there are many more pieces to add to this intricate puzzle, and the present findings suggest that an important piece may be how residents perceive diversity and segregation at local, regional, and national levels, and how this influences trust in all its complexity. Altogether, deeper reflections on the causes of (perceived) segregation is warranted (*cf.*, Banaji et al., 2021).

Finally, this study was conducted in the Netherlands, so a contextualization of our findings is required. Gesthuizen et al. (2010) confidently concluded that “Putnam’s hypothesis on ethnic diversity must be refuted in European societies” (p. 121). We would like to nuance this claim and are less eager to dismiss the issue as merely an “American problem” (see also Phillips, 2013). Indeed, also in European countries, Putnam’s pessimistic perspective may still hold for a part of the population, that is, those individuals that perceive a lot of diversity and a lot of segregation in their city. However, it is important to state in this context, unlike outgroup trust, ingroup trust is being unaffected by (perceptions of) diversity and segregation. This resonates with Burt (2002) finding that social capital needed for bridging with other groups is more difficult to achieve and requires more investment than social capital for bonding within one’s own group. That said, the interaction effects between diversity and segregation, both objectively and subjectively assessed, on several dimensions of social cohesion should be explored in other countries as well, also beyond the European continent. To conclude, Confucius’ adage was “May you live in interesting times.” While we undoubtedly do at the moment, we would like to add “May you perceive low segregation (and reap the fruits of contacts with neighbors who are different).”

## Data availability statement

The raw data supporting the conclusions of this article will be made available by the authors, without undue reservation.

## Ethics statement

Ethical review and approval was not required for the study on human participants in accordance with the local legislation and institutional requirements. The patients/participants provided their written informed consent to participate in this study.

## Author contributions

JVA, AVH, and AR developed the study concept and design. Data collection, data analysis and interpretation was conducted by JVA. JVA drafted the manuscript, and SAV, AR, and AVH provided critical revisions. All authors approved the final version of the manuscript for submission.

## Funding

This publication was made possible by a senior postdoctoral research grant from the Research Foundation - Flanders (FWO.3E0.2021.0085.01).

## Conflict of interest

The authors declare that the research was conducted in the absence of any commercial or financial relationships that could be construed as a potential conflict of interest.

## Publisher’s note

All claims expressed in this article are solely those of the authors and do not necessarily represent those of their affiliated organizations, or those of the publisher, the editors and the reviewers. Any product that may be evaluated in this article, or claim that may be made by its manufacturer, is not guaranteed or endorsed by the publisher.

## Footnotes

^1^There were on average 14 respondents per city (*SD* = 7.87). *Post-hoc* sensitivity analyses, conducted with the ‘pwr’ package (Champely et al., 2015) in R (version 3.3.1; R Core Team, 2015), indicated that our power to find a small-to-medium-sized interaction effect, given the sample size, was.82.

^2^These 50 cities differ widely in terms of proportion of people with a migration background living there. Note that this dataset has also been used in Study 1 of (Van Assche et al., 2018a), but only the actual diversity indicators and the trust scale overlap (i.e., no perceived diversity, actual segregation, or perceived segregation effects were examined in that study).

^3^For the sake of parsimony and clarity, we only present the results from these analyses here. Results from three integrative models testing the effects of actual diversity, perceived diversity, actual segregation, perceived segregation, their two-way interactions, three-way interactions, and the four-way interaction effect on general, ingroup, and outgroup trust yielded very similar results. Analyses excluding Rotterdam (i.e., the city with the highest actual diversity and segregation scores) also yielded almost identical results. Likewise, analyses controlling for age, gender, education, and income level (see also Dotti Sani and Magistro, 2016; Kim et al., 2022) yielded similar results, with two small exceptions: the main effect of perceived segregation on general trust (Model 1 and 4) and the interaction effect between actual diversity and perceived segregation on ingroup trust (Model 2) became marginally significant (see Appendix A for more detailed information).
